# Development of a Curved, Stratified, *In Vitro* Model to Assess Ocular Biocompatibility

**DOI:** 10.1371/journal.pone.0096448

**Published:** 2014-05-16

**Authors:** Cameron K. Postnikoff, Robert Pintwala, Sara Williams, Ann M. Wright, Denise Hileeto, Maud B. Gorbet

**Affiliations:** 1 Systems Design Engineering, University of Waterloo, Waterloo, Ontario, Canada; 2 Alcon/CIBAVision, Duluth, Georgia, United States of America; 3 School of Optometry and Vision Science, University of Waterloo, Waterloo, Ontario, Canada; Université de Technologie de Compiègne, France

## Abstract

**Purpose:**

To further improve *in vitro* models of the cornea, this study focused on the creation of a three-dimensional, stratified, curved epithelium; and the subsequent characterization and evaluation of its suitability as a model for biocompatibility testing.

**Methods:**

Immortalized human corneal epithelial cells were grown to confluency on curved cellulose filters for seven days, and were then differentiated and stratified using an air-liquid interface for seven days before testing. Varying concentrations of a commercial ophthalmic solution containing benzalkonium chloride (BAK), a known cytotoxic agent, and two relevant ocular surfactants were tested on the model. A whole balafilcon A lens soaked in phosphate buffered saline (BA PBS) was also used to assess biocompatibility and verify the validity of the model. Viability assays as well as flow cytometry were performed on the cells to investigate changes in cell death and integrin expression.

**Results:**

The reconstructed curved corneal epithelium was composed of 3–5 layers of cells. Increasing concentrations of BAK showed dose-dependent decreased cell viability and increased integrin expression and cell death. No significant change in viability was observed in the presence of the surfactants. As expected, the BA PBS combination appeared to be very biocompatible with no adverse change in cell viability or integrin expression.

**Conclusions:**

The stratified, curved, epithelial model proved to be sensitive to distinct changes in cytotoxicity and is suitable for continued assessment for biocompatibility testing of contact lenses. Our results showed that flow cytometry can provide a quantitative measure of the cell response to biomaterials or cytotoxic compounds for both the supernatant and adherent cell populations. As a specifically designed *in vitro* model of the corneal epithelium, this quantitative model for biocompatibility at the ocular surface may help improve our understanding of cell-material interactions and reduce the use of animal testing.

## Introduction

The cornea is comprised of three main cellular layers: the epithelium, stroma, and endothelium. The corneal epithelium is the first line of defence against many types of injury, trauma, and infection and contributes to maintenance of transparency and rigidity of the cornea [1]–[3]. The epithelium has also been shown to be the primary barrier against transcorneal permeation [4]. As a result, to have simpler models than the entire cornea itself, many researchers have opted to develop alternative corneal epithelial models for the study of material interactions at the front of the eye.

Recently, *in vitro* ocular toxicity testing has experienced a major advancement with the development of multilayered corneal epithelial cultures. There has been much interest in developing *in vitro* models that have the potential to replace the *in vivo* Draize test. The Draize test was developed as an ocular toxicity test in 1944 and involves the placement of test solutions on the eyes of living animals [5]. It became part of the United States Food and Drug Administration regulations in 1964 [6]. The Draize test has since come under much criticism in terms of its performance and reliability [6]–[8]. As the corneal epithelium represents the major barrier of the eye, many *in vitro* epithelial models have since been developed and proposed as alternatives to the *in vivo* Draize test. Since the 1960s, monolayer cell cultures have been developed using primary and immortalized corneal epithelial cell lines of rabbit and canine origin; and starting in the 1990s, the use of human-derived cells has become more popular [9], [10].

Concurrent to the development of better cell lines was the improved understanding of multilayered cultures and the importance of the air-liquid interface. Combined, the research has led to many *in vitro* models of epithelial reconstruction [11]–[14] and the development of commercially available models such as HCE by SkinEthic, EpiOcular by MatTek, and Clonetics by Lonza. The majority of these *in vitro* models have been used for toxicity testing of different ophthalmic solutions and for drug permeation studies [15]–[18], though presently none of these models have been validated or accepted for regulatory purposes. On the other hand, little research has been performed with these *in vitro* models in the area of contact lens interactions, possibly due to their limited surface area that would require the manufacture of smaller lenses or the use of trephined lens segments.

As part of regulatory testing, contact lenses are rigorously tested for their interactions with the ocular surface. Beyond the initial qualification of the contact lens material, the overall biocompatibility of a multipurpose contact lens disinfecting solution (MPS) with the contact lens material also needs to be defined. It is recognized that contact lens materials uptake and release some components of MPS, which may in turn affect ocular cell response [19]–[21]. Most notably, lens-solution interactions have been shown to be of critical importance in patterns of solution induced corneal staining as seen in both the StainingGrid and the IER Matrix Study [22], [23]. Current *in vitro* toxicological evaluations have been mostly conducted using a monolayer of cells to investigate the effects of different MPS [24]–[27] (see Choy *et al*. [27] for a review of these studies). In line with Powell’s recent demonstration that lens material has an effect on uptake and release of biocides [19], our research group further showed that this difference in uptake and release can affect the response of corneal epithelial cells *in vitro* [28], [29]. While monolayer cultures represent a rapid and simple model to investigate lens-solution interactions, these models are very sensitive as they solely consist of a single layer of cells and are static in nature. As a means to better characterize and understand lens-solution biocompatibility, we proposed the development of an *in vitro* reconstructed curved epithelium model that can better mimic interactions at the interface between the contact lens and corneal epithelial cells. To determine the sensitivity of the curved, stratified model, we chose to assess the response of our model to varying concentrations of benzalkonium chloride, a well-known cytotoxic agent [30], and compare that response to a silicone hydrogel lens soaked in phosphate buffered saline. As benzalkonium chloride is often found in a proprietary solution, common ophthalmic surfactants from the poloxamine family were also tested to ensure cytotoxicity was dependent on benzalkonium chloride.

To evaluate the response of the curved stratified epithelium, the MTT and luciferin-luciferase ADP/ATP assays were used to determine cellular metabolic activity and flow cytometry was performed to investigate changes in integrin expression and mechanism of cell death. To our knowledge, this current study reports the first *in vitro* stratified model developed specifically for studying contact lens-solution biocompatibility; this model may further prove useful for drug-delivery and cell-material interaction studies.

## Methods

### Reagents and Antibodies

Keratinocyte serum-free medium (KSFM), keratinocyte growth supplements (KGS), and penicillin/streptomycin (Pen/Strep) solution were purchased from ScienCell (Carlsbad, California). All other cell culture reagents, including Dulbecco’s Minimum Essential Medium (DMEM), 1∶1 DMEM in Ham’s F12 nutrient medium, fetal bovine serum (FBS), 0.25% Trypsin-EDTA, Hank’s based cell dissociation buffer, and TrypLE Express were purchased from Life Technologies (Burlington, Ontario, Canada). Phosphate buffered saline (PBS) was purchased from Lonza (Allendale, New Jersey).

Monoclonal antibodies to β_1_ integrin (CD29) and α_3_ integrin (CD49c) were fluorescein isothiocyanate (FITC) and R-phycoerythrin conjugated, respectively and were purchased from Becton Dickinson (Mountain View, CA, USA). Propidium iodide (PI) and the FLICA caspase kit were purchased from ImmunoChemistry Technologies, LLC (Bloomington, MN, USA). The FLICA kit uses the fluorescent probe FAM-VAD-FMK for caspase detection. The ADP/ATP Bioluminescent Cell Viability Kit II was purchased from PromoCell GmbH (Heidelberg, Germany). All chemicals used to prepare paraformaldehyde and HEPES Tyrode Buffer were of analytical or reagent grade.

### Contact Lens and Ocular Solution

A daily-wear silicone hydrogel balafilcon A (BA; Bausch & Lomb, Rochester, NY, USA) was tested. Lenses were obtained in their original packaging from the manufacturer and had a curvature of 8.6 mm, diameter of 14.0 mm, and power of −3.00 dioptres. All lenses were used before their expiry date. Whole lenses were used and were not cut before placement on the cultures.

Phosphate buffered saline was used as a negative control lens solution, which was previously shown to be biocompatible [31]. A sterile ophthalmic solution of benzalkonium chloride (BAK) was used as a positive control: the commercially available Moisture Eyes (ME; Bausch & Lomb, Rochester, NY, USA) has a BAK concentration of 0.01% w/v. Stratified cultures were exposed to undiluted and diluted Moisture Eyes at final BAK concentrations of 0.01% w/v (100% ME), 0.005% w/v (50% ME), and 0.002% w/v (20% ME).

As Moisture Eyes is a proprietary commercial source of BAK, it is possible that other ingredients within the solution may affect viability. To confirm that the cytotoxicity observed with our BAK source resulted mainly from BAK, two commonly used ocular surfactants from the poloxamine family, namely Tetronics 904 and 1304 (BASF, Ludwigshafen, Germany) were also investigated. Surfactants were tested at concentrations of 0.25% w/v and 1% w/v which are within a relevant range for ocular therapeutics [32], [33].

### *In vitro* Cell Culture

HPV-immortalized human corneal epithelial cells gifted from Dr. May Griffith [10] were cultured in keratinocyte serum-free medium with keratinocyte growth supplements (bovine pituitary extract and recombinant epidermal growth factor) and Pen/Strep. Fresh medium was added every other day, and cells were grown to 90% confluency and were used before their eighteenth passage. Higher passage numbers were avoided in order to reduce the genomic variations that have been observed in other immortalized epithelial cells [34]. Adherent cells were removed using TrypLE Express dissociation solution. Cells were routinely observed for any morphological changes.

### Curved Multilayer Preparation

**Figure 1:**
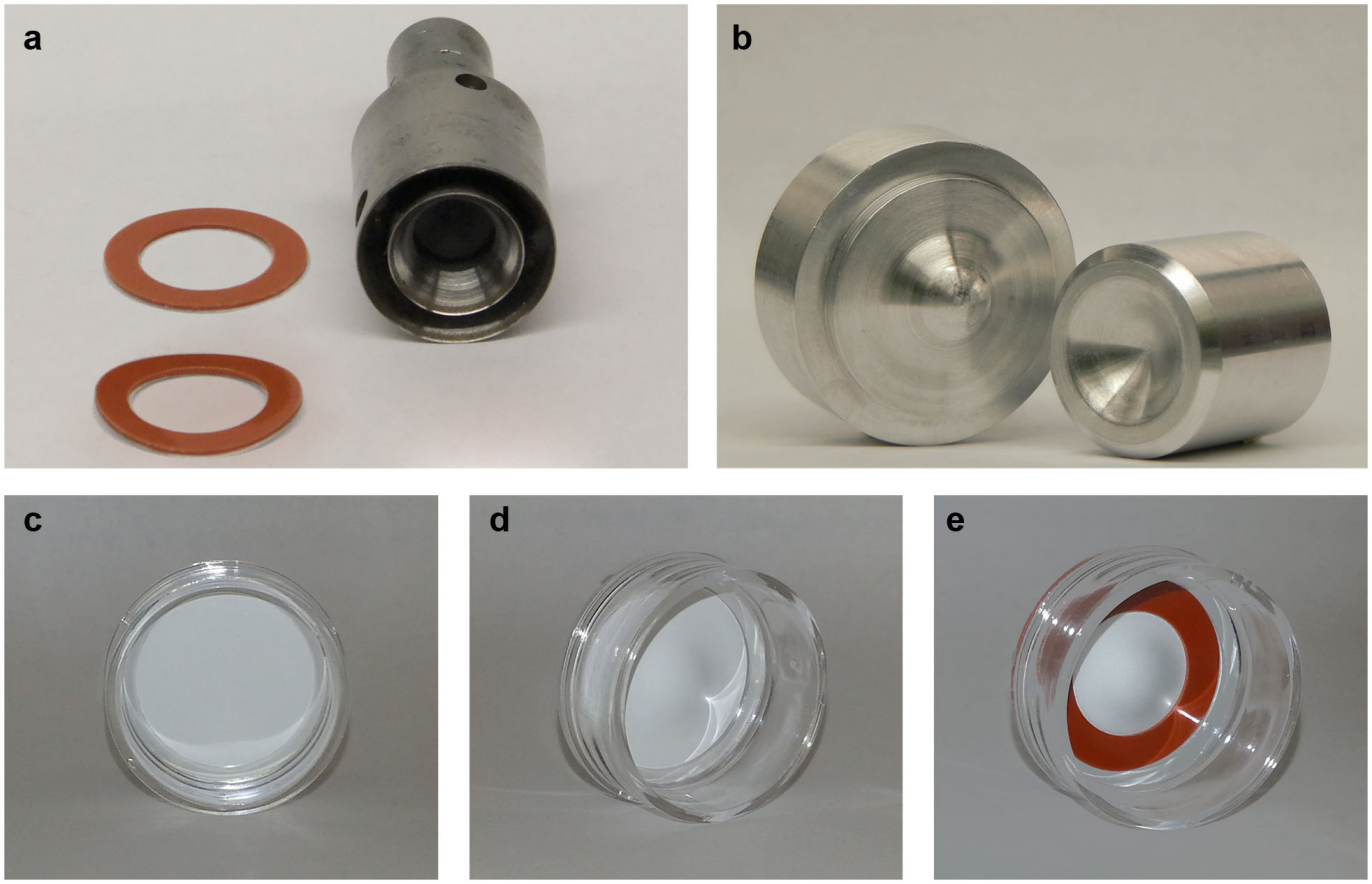
Necessary materials to build a curved cellulose insert for in vitro epithelial surface reconstruction. a) silicone rings and silicone ring die, b) top and bottom of eye form, c) cellulose insert before forming, d) final curved insert, e) final curved insert with one silicone ring.

The curved multilayers were grown on a Millicell-HA (mixed cellulose esters, Millipore, Billerica, MA, USA) membrane with a 0.45 µm pore size. To deform the cell substrate with a curve that would mimic the curvature of the cornea, an aluminum mold/counter-mold was fabricated (Figure 1b). The 30 mm diameter filters were first deformed using the custom-shaped curved die. Silicone Press-to-Seal sheets with adhesive (Life Technologies, Burlington, Ontario, Canada) were punched into rings (inner diameter of 15 mm and an outer diameter of 22.5 mm) using a laboratory bench press (Figure 1a). Once cut, the rings were disinfected with 70% ethanol and placed on top of the curved filters to reduce the cell culture surface area to the curved filter. The entire process is depicted in Figure 1 where the initial cellulose filter (Figure 1c) is deformed into a curved shape (Figure 1d) and finally, the ring is placed on top (Figure 1e). The assembled inserts were then UV sterilized. After sterilization, inserts were coated with collagen type I (0.05 mg/mL –30 min at 37°C) and cells were seeded within 30 minutes of coating.

The curved inserts were seeded with 5×10^5^ cells per insert. The curved multilayers were fed with KSFM on each of the basal and apical sides of the curve for the first seven days, with medium being exchanged every other day. After seven days, cell differentiation was induced by exposing the monolayer to an air-liquid interface. Cells were fed only on the basal side with 2% FBS in 1∶1 DMEM/F12 and medium was exchanged daily. The cells grew under these conditions for seven days and were then ready for experimentation.

### Histopathological Evaluation

Consecutive sections constituting the entirety of the specimens were processed routinely for microscopic examination: after initial fixation in 10% neutral buffered formalin, the cellulose filters were processed, embedded in paraffin, serially sectioned in 5-µm-thick sections, and stained with hematoxylin and eosin (H&E). The histological slides were evaluated using bright field microscopy (Leica DM1000, Leica Microsystems Inc, Concord, ON).

### *In vitro* Onlay Model

For the testing of solution soaked contact lenses, we adapted our monolayer onlay method[31] to be used in a stratified and curved epithelial model. Contact lenses were incubated in 3 mL of PBS for 24 hours in a tissue-culture-polystyrene well. Prepared stratified multilayers were fed with KSFM on the basal side. Apically, KSFM was added to wet the surface and PBS-soaked lenses were placed gently on top of the multilayer, concave-side down, to fit the eye-shaped curve. An additional amount of KSFM was added on top of the lens to ensure moisture retention. For the BAK samples, 800 µL, 400 µL, and 160 µL of BAK solution were added to the multilayers for the 0.01%, 0.005%, and 0.002% BAK dilutions, respectively. KSFM was added, where necessary, for a total apical volume of 800 µL. Tetronics 904 and 1304 were dissolved in PBS to a concentration of 2% w/v and were further diluted with KSFM to final concentrations of 0.25% and 1% on top of the stratified cultures. Control cells were fed with 800 µL of KSFM. Upon placement of the lenses, surfactants, and BAK, the cells were returned to the humidified incubator at 37°C and 5% CO_2_ for either 6 or 24 hours.

### MTT Assay

After 6 and 24 hours incubation, lenses and medium were removed to quantify cellular viability using the MTT assay. The MTT assay was originally developed in 1983 by Mosmann et. al. [35] based upon the reduction of tetrazolium salts as originally discovered by Altman [36], [37]. The assay was then redeveloped for multilayers by Doucet in 1996 [38], adapted again by Van Goethem in 2006 to improve accuracy [15], and finally reworked by Pauly to further improve apical sensitivity in 2009 [39]. To allow the MTT assay to be used for our larger and curved stratified model, Pauly’s protocol was further modified as follows. Briefly, thiazoyl blue tetrazolium bromide (0.5 mg/mL, MTT, Sigma Aldrich, Oakville, ON, Canada) was added to the apical and basal sides of the cell culture insert and was incubated for 3 hours 37°C and 5% CO_2_. The MTT solution was then removed and isopropanol was added to both the apical and basal sides of the insert and plates were agitated for 2 hours. An aliquot was taken from both the apical and basal sides and the two remaining solutions in the apical and basal sides were then mixed together and a final aliquot of the mixed solution was taken. Samples were read in a UV-Vis spectrophotometer for optical density at 595 nm with a reference at 650 nm. All results are expressed as relative viability compared to control cells; cells incubated in KSFM and in the absence of a contact lens (no lens control). Cellular metabolic activity determined from the mixed solution is reported in the results section.

### Cell Collection and Tissue Digestion

After lens incubation, the media on top of the well was used to gently wash the contact lens and the surface of the stratified cell culture. This population of cells and debris was collected and is referred to as the **supernatant** population. Upon removal of the supernatant population, DMEM/10% FBS was added to the supernatant cells and the samples were centrifuged. Supernatant cells were resuspended in DMEM/10% FBS and prepared for flow cytometry (see below).

Once the supernatant population and the contact lens were removed, the multilayers were digested. 0.25% Trypsin-EDTA was added to the basal side of the insert and to the top of the insert. Cell dissociation buffer was then added to the top of the insert and the cells were placed on a shaker at 120 rpm for 45 minutes in a 37°C and 5% CO_2_ environment. After 45 minutes, FBS was added to the inserts and cells were briefly resuspended via pipetting. The cells were returned to the shaker for another 45 minutes. Once the cells were removed, the cell-containing media from the top of the filter was transferred to polypropylene tubes containing DMEM/10% FBS. Cells were then centrifuged and resuspended in fresh media. This population is referred to as the **adherent** population.

### Luciferin-luciferase ADP/ATP Assay

To further assess cellular viability, the Bioluminescent Cell Viability Kit II (ADP/ATP) was used for the determination of a viability ratio of ADP/ATP. The assay was performed as per manufacturer’s instructions. Briefly, ATP monitoring enzyme and nucleotide releasing buffer were mixed to form a “Reaction Mix”. 100 µL of the Reaction Mix was added to a 96-well plate. This sample was then read on a luminescence-capable spectrophotometer (FluoStar Optima, BLG Labtech, Ortenberg, Germany) to provide the background reading (Data A). Digested adherent cells were then diluted and 5×10^3^ cells per 10 µL of medium were added to the 96-well plate. This cell solution was then read on the luminometer, twice (Data B and C) to measure the amount of ATP within the cells. Finally, 1 µL of ADP converting enzyme was added and the cell solution was read for a final time (Data D). To obtain the ratio of ADP to ATP, the following calculation was used:
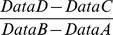



All results are expressed as ratios relative to control cells: cells incubated in KSFM in the absence of a contact lens (no lens control).

### Flow Cytometry

After resuspension in DMEM/10% FBS, aliquots were transferred to flow cytometry tubes. Flow cytometry tubes contained one of two mixtures: (a) FITC-labeled anti-CD29 and PE-labeled anti-CD49c; or (b) FITC-VAD-FMK (pan caspase). Integrins were incubated in the dark at room temperature for 30 minutes. Integrin samples were then diluted in buffer and paraformaldehyde fixative (1% final concentration) and were analyzed by flow cytometry within five days. As per kit instructions [40], caspase samples were incubated at 37°C for 60 minutes. The samples were then washed and propidium iodide (PI) was added. All caspase samples were analyzed immediately on the flow cytometer.

### Flow Cytometry Acquisition/Analysis

All integrin, caspase, and PI samples were acquired on a FACSVantage flow cytometer (Becton Dickinson, Mountain View, California) using CELLQuest Software. Appropriate isotype controls were used with each experiment. At least 5000 events were collected per sample. Analysis was also performed using FACSExpress post data acquisition.

### Statistical Analysis

All results are reported as means ± standard deviation. To evaluate the significance of the differences in cell viability and cell activation, an analysis of variance (ANOVA) was performed, followed by multiple pairwise comparisons using the Tukey’s Honest Significant Difference using Statistical Analysis Software (SAS, Cary, NC, USA). Samples were compared to cells incubated without lens or solution (no lens control) as well as to cells exposed to a PBS-soaked BA lens (PBS-BA control). Significant differences between the compounds tested are also reported. A *p* value of less than 0.05 was required for statistical significance. The number of experiments was equal to or greater than three and experiments were performed on different days.

## Results

**Figure 2:**
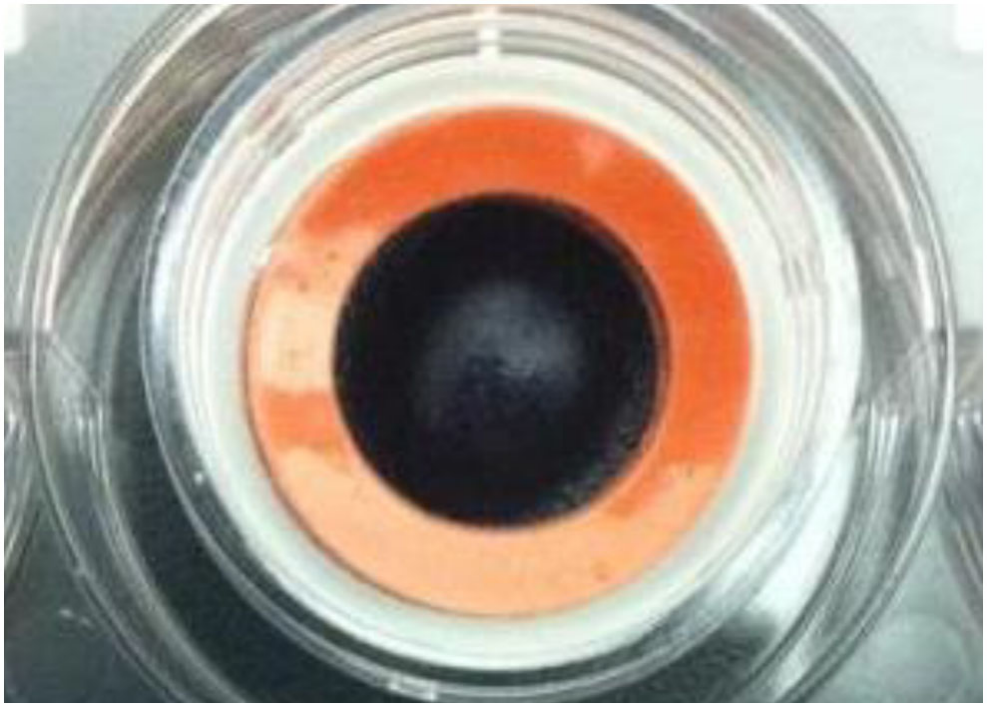
Curved stratified epithelium following exposure to MTT. Surface coverage by formazan indicates well stratified and viable cells.

While a cellulose insert can be curved and hold its shape, one of its drawbacks is that it is not transparent, and thus difficult to image. To illustrate how cells cover the entire curved surface, pictures were taken after incubation with the metabolic dye thiazoyl blue tetrazolium bromide (MTT) (Figure 2).

**Figure 3:**
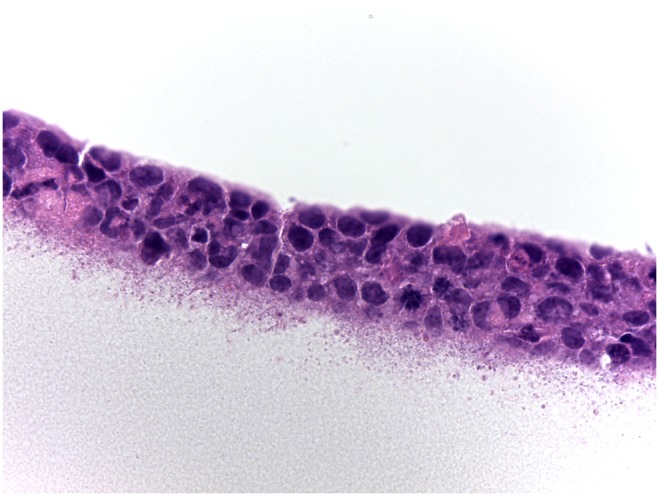
Squamous epithelial cells arranged in layers, firmly attached to the underlying cellulose filters. H&E, original magnification 63X.

Histopathologic evaluation revealed squamous epithelial cells arranged in 3 to 5 defined, relatively uniform horizontal layers, firmly attached to the underlying cellulose filter (Figure 3).

### Cellular Metabolic Activity

**Figure 4:**
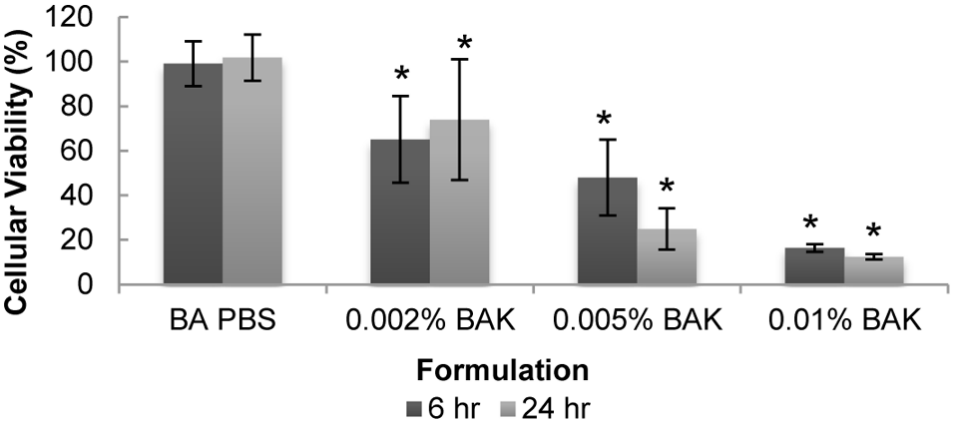
Effect of BAK and PBS-soaked lens on cellular viability of curved stratified epithelium. Cells were incubated for 6 and 24 hours with solutions of BAK or PBS-soaked contact lenses. Viability was measured by the MTT assay and is expressed as a percentage relative to cells grown in the absence of a contact lens (control). n = 5, *significantly different from BA PBS control, p<0.05. BA PBS: PBS-soaked balafilcon A lens.

As shown in Figure 4, there was no difference in viability between the BA PBS (99±10% and 102±10% at 6 and 24 hours, respectively) and the no lens controls. All BAK concentrations significantly reduced viability when compared to the BA PBS control (p<0.001). Exposure to 0.01% BAK resulted in the largest reduction in viability with values of 16% and 12% at 6 hours and 24 hours, respectively. Time was not a significant factor (p = 0.74) suggesting that any of the tested concentration of BAK could be used as a positive control at either time point.

**Figure 5:**
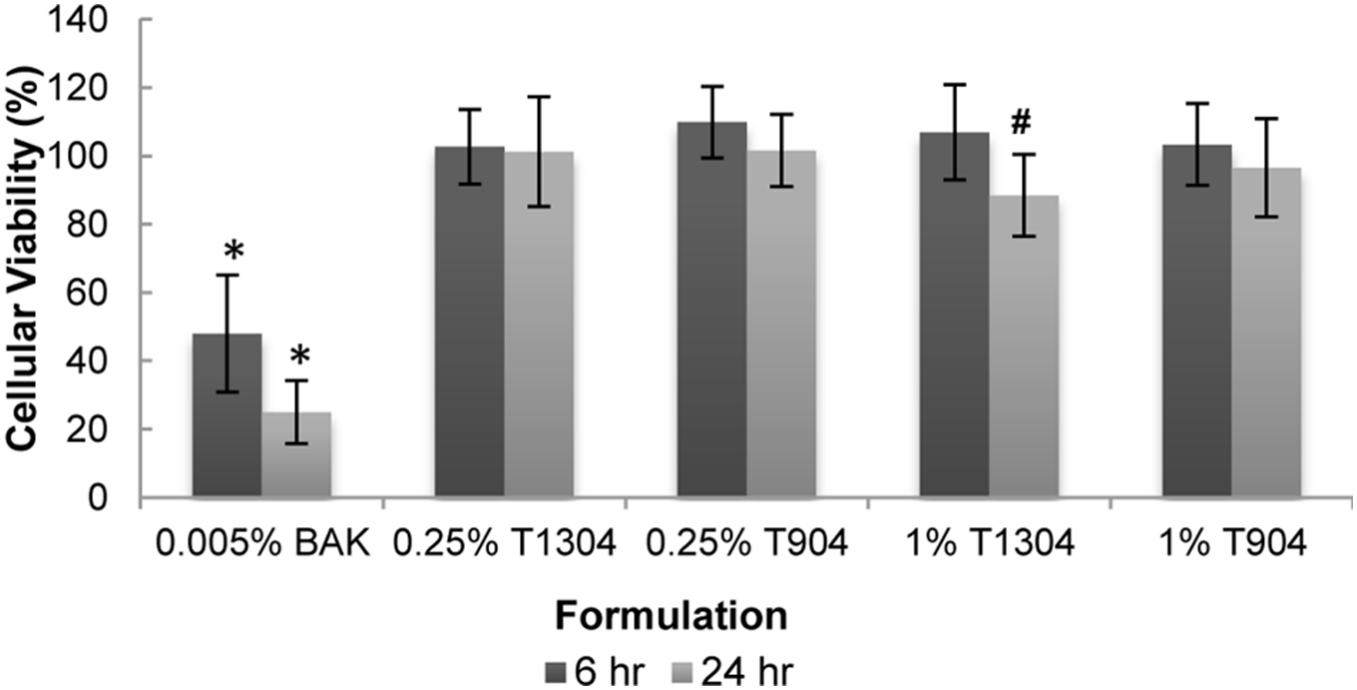
Effect of ocular surfactants on cellular viability of curved stratified epithelium. Cells were incubated for 6 and 24 hours with solutions of different poloxamine surfactants at different concentrations. Viability was measured by the MTT assay and is expressed as a percentage relative to cells grown in the absence of a contact lens (control). n = 5, *significantly different from BA PBS control, ^#^significantly different between 6 and 24 hours, p<0.05, T1304: Tetronic 1304, T904: Tetronic 904.

Both surfactants tested showed a negligible reduction in viability at 6 hours (Figure 5). Only exposure to 1% Tetronic 1304 for 24 hours resulted in a significant decrease in viability (88±12%) when compared to the viability observed at 6 hours (107±14%) (p<0.05). However, this reduction in viability was not statistically significant when compared to the BA PBS control (p = 0.14).

### ADP/ATP Viability Ratio

With the luciferase assay, when compared to controls (BA PBS and no lens), an increase in the ratio of ADP/ATP was observed with increasing concentrations of BAK, except in the case of 0.01% BAK (Table 1). It is important to note that due to the damage incurred from exposure to the most toxic concentration of BAK (0.01%), cells from this sample may have oversaturated the signal, which resulted in a calculated ADP/ATP ratio that appeared to be non-significantly different from the controls (Personal communication, 2014, Jacqueline White, PromoCell).

**Table 1:** Effect of surfactant, BAK, and PBS-lens exposure on the metabolic activity of corneal epithelial cells in a curved, stratified culture.

	Average	Stdev
**BA PBS**	1.03	0.65
**1% T1304**	1.32*	0.66
**0.002% BAK**	1.47*	0.38
**0.005% BAK**	2.69*	1.37
**0.01% BAK**	1.10	0.42

Following exposure to the stimuli for 24 hours, the stratified culture was digested and the cells were routinely processed for ADP/ATP expression using a luciferase assay. All values are expressed as a ratio relative to the ADP/ATP ratio on cells incubated with no lens or solution (no lens control).

n = 4.

*significantly different from BA PBS control, p<0.05.

### Integrin Expression

To assess if exposure to BAK or to a PBS-soaked lens affected the cell adhesion phenotype, expression of the heterodimer α_3_β_1_ was investigated via its two integrin subunits α_3_ (CD49c) and β_1_ (CD29).

Two distinct populations of cells were studied. The supernatant population corresponds to the cells that were sloughed off of the multilayer during and after lens incubation or BAK exposure, and the adherent population refers to the tissue that was digested via trypsinization.

**Figure 6:**
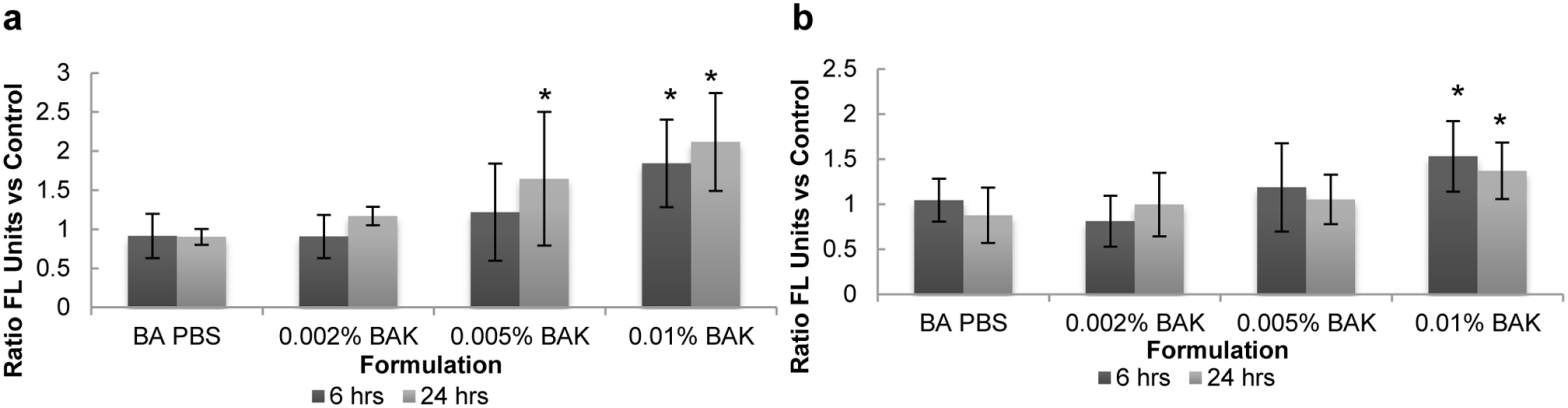
Effect of BAK exposure and PBS-soaked lens on (a) CD49c and (b) CD29 integrin expression. Following exposure to stimuli after 6 or 24 hours, the curved, stratified cultures of corneal epithelial cells were digested with trypsin and EDTA to form the adherent cell population. CD49c and CD29 expression was measured by flow cytometry and is expressed as a ratio relative to its expression on cells incubated with no lens or BAK (control). n = 3 to 5, *significantly different from PBS-soaked lens, p<0.05. BA PBS: PBS-soaked balafilcon A lens.

In the adherent cell population (Figure 6), when compared to both a PBS-soaked lens and control (no lens, no solution), upregulation in CD49c expression was observed with increasing concentrations of BAK. Exposure to 0.01% BAK for both 6 and 24 hours resulted in a significant upregulation of CD49c expression when compared to all other samples (p<0.001). At 24 hours, higher CD49c expression was also observed with 0.005% BAK (p<0.01). While for all BAK samples, increases in CD49c expression appeared to occur between 6 hours and 24 hours, these changes were not statistically significant (p>0.2).

CD29 expression on adherent cells, as shown in Figure 6, was also upregulated in the presence of BAK. Similar to CD49c expression, a significant upregulation could be observed following exposure to 0.01% BAK for 6 and 24 hours compared to BA PBS (p<0.001). No change in CD29 expression was observed for BA PBS. There was also no effect of time on the expression of CD29.

For cells in the supernatant, as shown in Table 2, no difference in CD49c expression could be observed amongst any of the concentrations of BAK versus the BA PBS and no lens controls. Incubation time also had no effect. Regardless of treatment, there was also little difference in CD29 expression on cells from the supernatant. Some downregulation (albeit not significant) was observed at 6 hours for both 0.005% BAK and 0.01% BAK. However, at 24 hours, for all samples, a significant upregulation in CD29 expression was observed compared to 6 hours (p<0.05).

**Table 2:** Effect of BAK exposure and PBS-soaked lenses on cell integrin expression in a curved, stratified culture of corneal epithelial cells after 6 and 24 hours.

Supernatant	CD49c				CD29			
	Average		Stdev		Average		Stdev	
	6 hrs	24 hrs	6 hrs	24 hrs	6 hrs	24 hrs	6 hrs	24 hrs
**BA PBS**	0.92	1.06	0.43	0.09	0.99	1.30*	0.18	0.40
**0.002% BAK**	0.76	0.84	0.23	0.14	0.95	1.09*	0.29	0.15
**0.005% BAK**	0.99	0.72	0.57	0.40	0.78	1.00*	0.39	0.46
**0.01% BAK**	0.78	0.84	0.31	0.20	0.79	1.23*	0.31	0.32

Following exposure to stimuli, the surface of the stratified cultures were gently washed and this supernatant was collected and processed. CD49c and CD29 expression was measured by flow cytometry and is expressed as a ratio relative to expression on cells incubated with no lens or BAK (control). BA PBS: PBS-soaked balafilcon A lens.

n = 3–5.

*significantly different from 6 hour value, p<0.05.

### Caspase Activation and Propidium Iodide Expression

To assess how the different conditions affected cell apoptosis and necrosis, caspase activation was detected by flow cytometry using the fluorescently-tagged pan caspase inhibitor FITC-VAD-FMK, which fluoresces most intensely in cells with active caspases. Dead cells were identified by propidium iodide staining.

**Figure 7:**
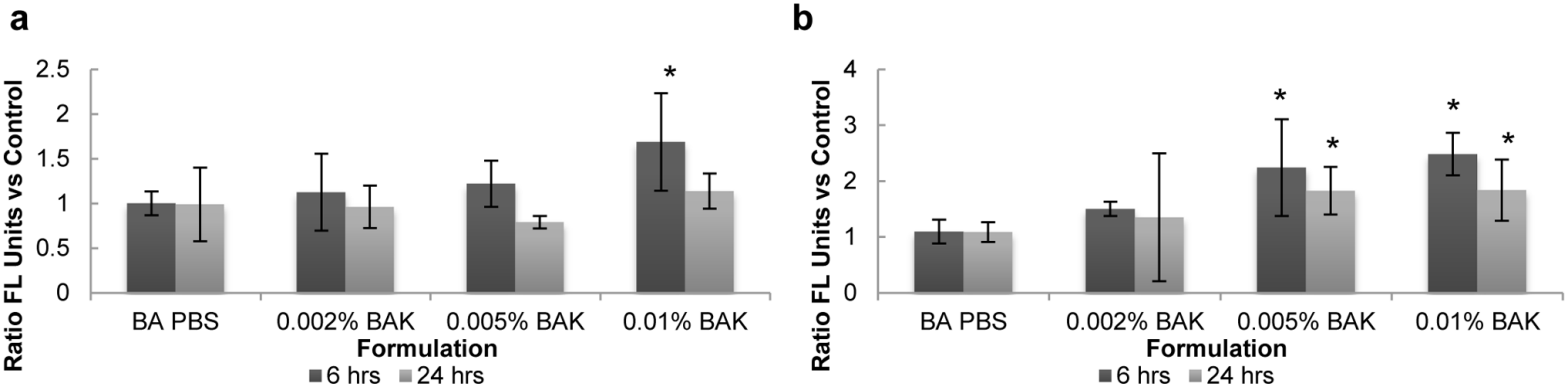
Effect of BAK exposure and PBS-soaked lens on caspase expression in the (a) adherent and (b) supernatant populations. Following exposure to stimuli after 6 or 24 hours, the curved, stratified cultures of corneal epithelial cells were digested with trypsin and EDTA. Caspase expression was measured by flow cytometry and is expressed as a ratio relative to its expression on cells incubated with no lens or BAK (control). n = 3 to 5, *significantly different from PBS-soaked lens, p<0.05. BA PBS: PBS-soaked balafilcon A lens.

For adherent cells, no difference in caspase activation was observed between BA PBS and the no lens control with ratios of 1.0±0.1 and 0.99±0.5 for 6 hours and 24 hours, respectively, as shown in Figure 7. The only formulation to induce significant caspase activation was 0.01% BAK at 6 hours (p<0.001). Caspase activation in cells from the 0.01% BAK sample at 6 hours was also significantly higher compared to the 24 hour time point, with ratios of 1.7±0.5 and 1.1±0.2, respectively.

For the supernatant cells, incubation with BA PBS led to caspase activation similar to the no lens control at both 6 hours and 24 hours (Figure 7). For 0.005% BAK and 0.01% BAK, a significant increase (p<0.001) in the level of caspase activation was observed in the supernatant cells for both 6 and 24 hours.

The fluorescent intensities of caspase activation for the control show that, when compared to adherent cells, the supernatant cell population exhibited a significantly higher level of caspase activation (p<0.001) at the 6 hour time point with values of 4.10±1.0 and 8.48±3.6, respectively. At 24 hours, minimal difference in caspase expression was observed between adherent and supernatant cell populations for all samples.

**Figure 8:**
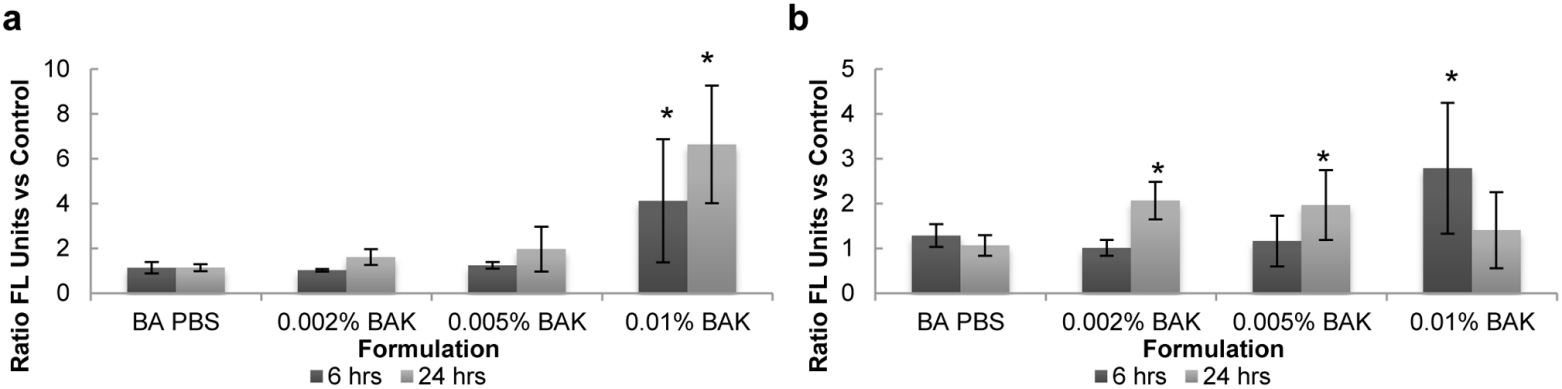
Effect of BAK exposure and PBS-soaked lens on PI staining in the (a) adherent and (b) supernatant populations. Following exposure to stimuli after 6 or 24 hours, the curved, stratified cultures of corneal epithelial cells were digested with Trypsin and EDTA. PI staining was measured by flow cytometry and is expressed as a ratio relative to its expression on cells incubated with no lens or BAK (control). n = 3–5, *significantly different from PBS-soaked lens, p<0.05.

In the adherent cell population, as expected, exposure to BAK led to an increase in cell death as shown by the increase in PI staining. At both 6 and 24 hours, PI staining in cells exposed to 0.01% BAK was significantly higher (p<0.001) compared to all other treatments (Figure 8).

**Figure 9:**
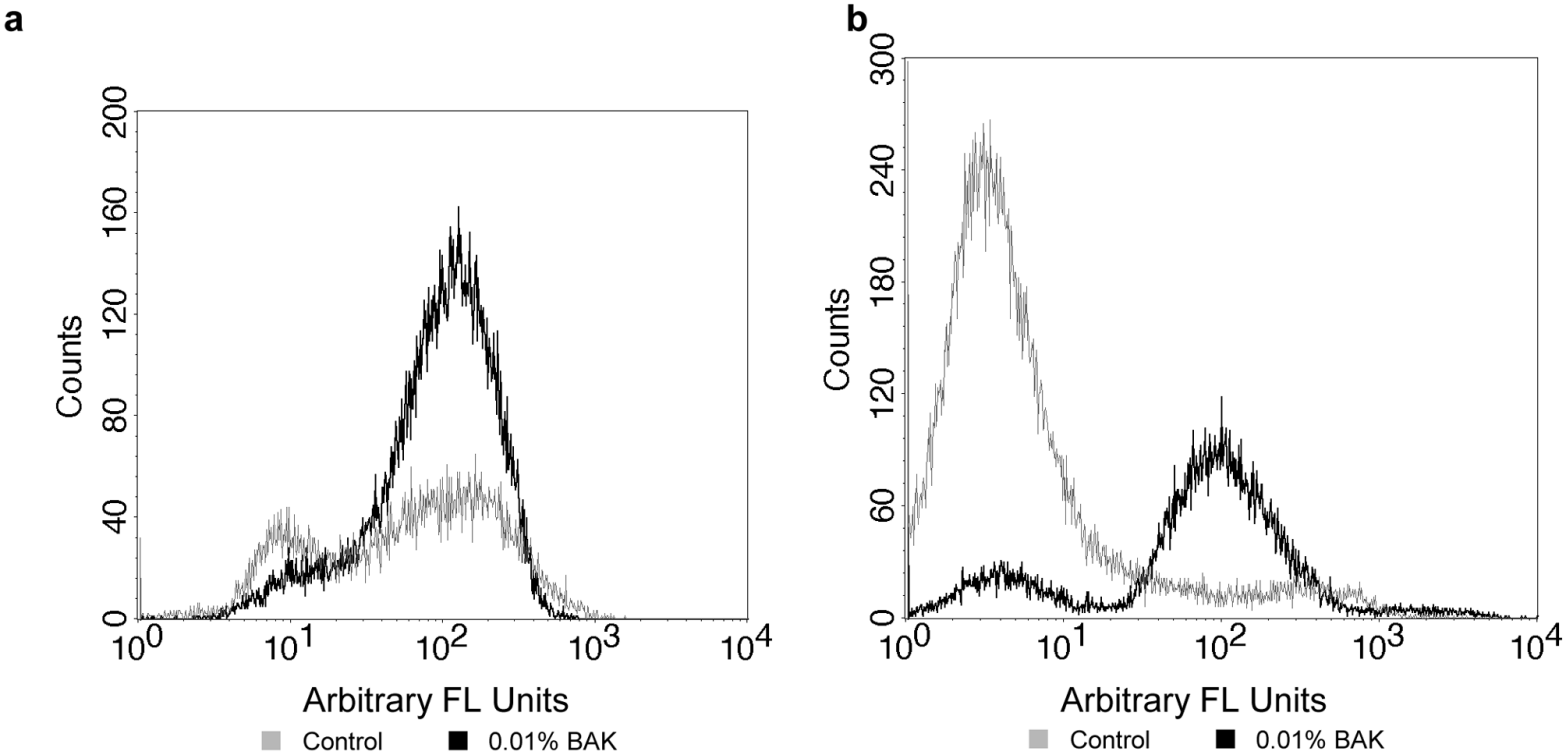
Cell death following BAK exposure as measured by PI staining. The histograms display PI staining in the supernatant (a) and adherent (b) cell population following 24 hour exposure to 0.01% BAK. The grey histogram represents cells from the no lens, no solution control; and the black histogram represents cells exposed to BAK. The positive peak for PI (>10 arbitrary fluorescent units) can be observed in both the control and BAK supernatant population, as cells slough off the stratified culture. A significant increase in cells staining positive for positive for PI can be observed following BAK exposure.

In the supernatant cell population, following exposure to BAK, a difference in PI staining was present between the 6 and 24 hour time points (Figure 9). At 6 hours, 0.01% BAK was the only formulation that induced a significant level of cell death. At 24 hours, both 0.002% BAK and 0.005% BAK led to a significant increase in PI staining in supernatant cells (p<0.05). For cells exposed to 0.01% BAK, a reduction in PI staining was observed between 6 and 24 hours (p<0.001).

In comparing the adherent versus supernatant population of cells in our no lens control, levels of PI staining were found to be significantly higher in the supernatant population (p<0.001), as shown in Figure 9. The adherent population of cells appeared to have a very low necrotic population in control samples, but the overall profile changed dramatically in response to 0.01% BAK where there were significantly more PI positive cells. This is in agreement with the MTT viability data.

## Discussion

### *In vitro* Model

Many other multilayered epithelial models exist, with the most popular commercial model being the SkinEthic Human Corneal Epithelial model that was developed in 2003 by Nguyen *et al* [41]. There has since been much use of the SkinEthic model to investigate the toxicity potential of ophthalmic solutions [39], [42]–[47] including a pre-validation study by Van Goethem *et al*. in 2006 where the SkinEthic model was shown to be a useful alternative for the *in vivo* Draize test [15].

The previously reported multilayered models have been grown using other cell types and support membranes: immortalized human corneal epithelial cell lines CEPI 17 CL4 and SV40 immortalized cells have been grown on polyester/polycarbonate Transwell cell culture inserts [48]–[51] and human corneal limbal epithelial cells have been grown on polyethylene terephthalate (PET) membranes [12]. Gipson [13] and Robertson [11] have also recently developed multilayered cultures. Finally, tissue reconstruction for corneal transplantation has given rise to many different multilayered models with cells grown on human amniotic membrane or silk fibroin [52]–[54].

To the best of our knowledge, none of the prior models have been focused on the investigation of contact lens interactions on a curved multilayered epithelial substrate. A cellulose-based filter was chosen as the substrate upon which the cells would grow. These filters are capable of plastic deformation and are neither too rigid nor brittle for our purposes. Contact lenses also fit very well to the shape of the curve. The main disadvantage of using a cellulose support is its opacity, as this makes it very difficult to image. However, the MTT assay has shown to be a very useful indicator of cellular health as indicated by formazan coverage across the filter surface.

Two major implementations used in the development of the curved multilayered epithelium were the use of a collagen coating and the use of an air-liquid interface. The basement membrane, between the corneal stroma and epithelium, is a specialized extracellular matrix supporting the corneal epithelium. It is composed mainly of laminin, collagen type IV, and heparin sulphate proteoglycan (perlecan) [55]. Human corneal epithelial cells grown on collagen gels are known to secrete all of the components of the basement membrane, as well as collagen type VII: a main component of anchoring fibrils which help epithelial cells attach to the stroma [55]. The use of collagen as a matrix has been shown to support attachment, proliferation, and differentiation of corneal epithelial cells [50], [56]–[58]. Collagen has also been shown to have a very low antigenicity [57].

Since the *in vivo* epithelium is exposed to air, it is a logical assumption that the main parameter affecting multilayer stratification and growth is the culturing of cells using an air-liquid interface. It has been shown that the air-liquid interface not only promotes proliferation, stratification and differentiation of corneal epithelial cells [56], [59], but it is actually essential for growth of a properly stratified epithelium [60]–[62]. It is believed that the air-liquid interface promotes the shift of oxidative metabolism from the growth phase to the differentiation phase [60]. Further, it has been shown that an air-liquid interface encourages the deposition of type VII collagen and laminin [59]. The air-liquid interface thus provides a polarization effect that allows for proper stratification. Upon the introduction of an air-liquid interface, our HCEC were able to properly stratify on the curved cellulose filter.

Histological analysis confirmed that our cells were stratified to three to five layers. The curved multilayer model thus offers an *in vitro* model whereby cells can be exposed to an entire soaked lens as if the model “wears” the lens, providing a better approximation of material interaction as it occurs *in vivo*. It remains, however, limited in its ability to fully mimic the *in vivo* environment as it does not account for tear film interactions, blinking, and inflammation; all of which may affect material biocompatibility. This model also does not account for other clinical implications of toxicity such as corneal edema, blisters, corneal opacity, and neovascularization [63]. In the current study, as a proof of concept and to ensure the reliability of the model, a PBS-soaked balafilcon A lens was used. As expected, the PBS-soaked lens proved to be very biocompatible with levels of cellular activity, integrin expression, and cell death, all comparable to a no lens control.

Previous work with stratified cultures has also focused on the viability and histology of the stratified corneal epithelium. To our knowledge, this is the first study that investigates differences in integrin expression and cell death in a stratified culture of corneal epithelial cells using flow cytometry. With the curved *in vitro* model, not only were the adherent cells that comprise the majority of the tissue characterized, but also the “shed” cells, which were collected from the culture media on the top of the filter prior to trypsinization. Digestion of *in vivo* corneal epithelial tissue for the purpose of flow cytometry has been done previously to study the necrotic population of cells [64]. *In vitro* stratified cultures have also been digested to examine the apoptotic response of corneal epithelium to UVB through colorimetric and fluorometric analyses [65].

### Cellular Viability

As previously described, the MTT assay is based on the reduction of tetrazolium salts. Mitochondrial, cytosolic, and microsomal enzymes are all capable of performing the reduction of MTT [66]. MTT is endocytosed into the cells where it is reduced to formazan in the endosomal/lysosomal compartment [67]. The formazan then accumulates in blue cytoplasmic granules and is exocytosed as needle-like aggregates on the cell surface [67], [68]. We therefore describe the MTT assay as a viability assay, which lends itself to a belief that it is a relative number of living cells to those that are dead. However, it may be more descriptive to refer to it as an assay of cellular activity. With increasing concentrations of BAK, cellular activity is greatly reduced at both 6 and 24 hours. Cells exposed to concentrations of 0.01% and 0.005% BAK sustained significant damage and continuous exposure to this dose of BAK prevented cell repair and growth. However, cells exposed to 0.002% BAK, while having a reduced viability at 6 hours, appeared to have a somewhat increased viability at 24 hours which would suggest that the stratified cells were able to repair themselves from the damage caused by BAK.

Our MTT viability results with BAK are in agreement with previously reported toxic mechanisms *in vitro*, primarily with the use of the SkinEthic model in the work of Baudouin and Khoh-Reiter [39], [42], [45]–[47]. Consistently, increasing concentration and time exposure to BAK is shown to have an increasingly negative effect on the health of corneal epithelial cells. However, our curved model appears to be slightly more sensitive to cytotoxicity than what has been shown using the SkinEthic model [39]: for similar levels of BAK exposure, approximately 20% to 60% viability was observed in our model compared to the SkinEthic model. These differences in viability may be due to differences in surface area, exposure time, BAK source, and other factors such as the different structure of the *in vitro* models.

The luciferase assay was originally developed using the biomimetic system found in fireflies [69]. Briefly, luciferase acts as an enzyme to catalyze the reaction between ATP and luciferin to form light: this light can then be measured using a luminometer. ADP can also be measured through its conversion to ATP and its subsequent reaction with luciferase. The ratio of ADP/ATP has since been shown to correlate with apoptosis and necrosis and to provide measures of cellular viability [70], [71]. Geerling *et al* [72] previously used a luciferin-based assay to assess the cytotoxicity of BAK using a monolayer model of primary corneal epithelial cells. The assay showed to be more sensitive than scanning electron microscopy evaluation of morphological changes as well as the live/dead calcein/ethidium homodimer fluorescence assay. Our luciferase results further confirmed our cellular viability findings as measured by MTT, namely an increase in cell death with increasing concentrations of BAK, demonstrating that the luciferase assay may also be used to detect cytotoxicity in stratified cultures.

### Poloxamine Surfactant Cytotoxicity

Poloxamines represent a family of nonionic amphiphilic surfactants and have been reported to be relatively nontoxic by BASF, their manufacturer [73]. Poloxamines are currently used in ophthalmology in contact lens cleaning solutions and eye drop formulations [32], [33], [74]. In our model, these surfactants show a negligible decrease in viability which is in accordance with recent *in vitro* and *ex vivo* corneal assays that demonstrated the safety of these surfactants for the ocular surface [75]. Poloxamers, similar to poloxamines but with a generally lower molecular weight, are also frequently used in ophthalmic preparations. While both families of surfactants have been shown previously to induce cytotoxicity *in vitro* [74], the study used a colony-forming assay of Chinese hamster lung fibroblasts V79 cells and a 6-day incubation. The long incubation time, the type of culture (isolated colonies versus stratified culture), and the cells used (V79), which may be more sensitive due to tissue and species variations, likely explain the difference in results. Overall, only 1% Tetronic 1304 showed a marginal decrease in viability (88±12% relative to control) as well as an increase in ADP/ATP relative to control, which may indicate some associated cell death. However, given the level of change observed compared to control, this concentration would likely not be considered cytotoxic.

### Integrin Expression

Integrins are heterodimeric transmembrane glycoproteins that consist of an alpha and a beta chain, and are responsible for the interactions between epithelial cells and their extracellular matrix. Integrin chains α_3_ (CD49c) and β_1_ (CD29) form heterodimers that are known to have strong roles in epithelial adhesion and maintenance of cell-cell junctions; they have also been reported to play a role in cell spreading [28], [76]. The heterodimer α_3_β_1_ is a receptor for fibronectin, laminin 5, laminin 10, and all major components of the corneal basement membrane [76] and α_3_β_1_ heterodimers are expressed in the basal or suprabasal layers of the corneal epithelium [77], [78]. Higher levels of α_3_β_1_ are expressed on cells with higher proliferative properties [77], [79], [80]. It has also been reported that β_1_ integrins can at least partially compensate for the loss of desmosomes and adherens junctions [77].

Integrin expression in supernatant cells appeared to be relatively consistent regardless of applied formulation and showed levels of expression similar to control cells. As the supernatant cells have been sloughed or worn off the multilayered tissue, it is likely that these cells no longer attempt to promote attachment. On the other hand, with increases in BAK concentration, the adherent cells showed a significant increase in CD49c (α_3_) and CD29 (β_1_) expression. This increase in integrin expression suggests that prolonged exposure to a cytotoxic stimulus such as BAK may lead the cells in the stratified tissue to enter a repair phase and consequently display a phenotype that increases their ability to proliferate and migrate.

### Cell Death Mechanisms

Apoptosis is a programmed form of cell death and serves, among others, as a defense mechanism in the removal of damaged cells. Apoptosis is a highly regulated, programmed cell death pathway, mediated in part by the action of caspases [81]. Caspases are cysteine aspartate proteases that act as mediators for initiating cellular disassembly and are normally found in the inactive, pro-caspase form [82], [83]. Initiation of apoptosis occurs upon activation of initiator caspases by intrinsic or extrinsic factors, which can subsequently activate downstream effector caspases.

Alternatively, necrosis is a form of dysregulated cellular death. Necrotic cells that have an intact nuclear membrane are stained by propidium iodide, which intercalates with DNA and provides a strong fluorescent signal in its bound form. It is also possible that cells positive for PI staining may be the result of an apoptotic death pathway.

Fluorescent levels of caspase and PI were much higher in the supernatant overall as compared to the adherent populations. This agrees with Pauly’s previous findings that cell death was localized to the outer layers of the multilayer following exposure to BAK [84].

Similar trends in caspase expression were observed in both supernatant and adherent cells whereby increases in BAK concentration resulted in increased caspase activation at 6 hours; albeit by 24 hours, caspase levels were lower compared to 6 hours. This is likely due to the 24 hour time point being too late to observe caspase activation. Caspase activation is an initiator of apoptosis and activation may not be detectable at later time points depending on the type of death cycle the cells may have entered (secondary necrosis versus apoptosis). Interestingly, the response of the multilayer to a strong stimulus (0.01% BAK) was different from a weak stimulus (0.002% or 0.005% BAK), suggesting a dynamic cell death process within the stratified culture model. With the 0.01% BAK samples, the reduction in PI staining between 6 and 24 hours in the supernatant was paralleled by an increase in PI staining in the adherent population. Thus, prolonged exposure to BAK may cause cells in the supernatant to lose their nuclear membrane integrity and ability to stain with PI: these cells detached from the stratified culture and thus were potentially already damaged, making them more sensitive to cytolytic damage by BAK. Such a phenomenon does not appear to occur at lower BAK concentrations as an increase in PI staining in supernatant cells is observed for these treatments at 24 hours. The change in PI expression may be explained by the apoptotic cells in the supernatant population entering secondary necrosis, which has been previously observed [64], [85]. The increase in PI staining at 24 hours for all BAK samples highlights the completion of the cell death cycle whereby a certain number of cells in the stratified culture were irreversibly damaged by exposure to BAK. The results from the 6 hour time point suggest that apoptosis played a role in the cell death observed at 24 hours. These results emphasize the importance of early time points when measuring cell death mechanisms. The PI staining results also correspond well with the observed reduction in viability as measured by MTT and the luciferase assay.

It is also worth discussing the overall lack of significant change observed with exposure to 0.002% BAK. While viability, as measured by MTT, decreased at both 6 and 24 hours, no significant upregulation of integrins or caspase expression occurred. PI staining increased in the supernatant cell population, but only after a 24 hour exposure. This was also noted for 0.002% BAK using the luciferase assay. This suggests that BAK may only be harmful at high concentrations: though cells may still be damaged, they may be able to withstand certain low concentrations of BAK, allowing the tissue to recover. These results are in accordance with the previous study by Pauly *et al*. [39] showing that cells exposed to lower concentrations of BAK did not see a continued decrease in reduction of viability following a 24 hour recovery period.

## Conclusions

A healthy, reconstructed corneal epithelium in the shape of the regular human cornea *in vitro* was successfully grown. Our model appears to be well-stratified with three to five layers of cells. The overall suitability of our model for biocompatibility experiments was verified by exposure to the well-known cytotoxic compound benzalkonium chloride, which is currently used as a preservative in commercially-available eyedrop formulations. Surfactant cytotoxicity was also found to not be implicated in the cytotoxicity of BAK.

To assess cellular viability and cell phenotype via flow cytometry, new protocols were developed for the stratified curved models. Both supernatant cells, which had been sloughed off of the multilayer, and adherent cells, which had remained adherent as part of the stratified culture, were characterized. Cells in the supernatant were found to be significantly more caspase and PI positive. A dynamic cell death mechanism was observed across the varying concentrations of BAK, leading to the hypothesis that many cells die through apoptosis. An increase in integrin expression was also noted with increasing concentrations of BAK implying that the cells were attempting to repair the damaged tissue. Examination of cell phenotype via flow cytometry proved to be a very useful quantitative method for detecting sensitive changes in our curved, stratified corneal epithelium.

Overall, our model was able to detect differences in cytotoxicity over time. Furthermore, exposure of the curved stratified culture to a PBS-soaked lens did not have a significant effect on cells, suggesting that our *in vitro* model is suitable to investigate contact lens/solution interactions.
